# The mediating role of coping strategies between depression and social support and the moderating effect of the parent–child relationship in college students returning to school: During the period of the regular prevention and control of COVID-19

**DOI:** 10.3389/fpsyg.2023.991033

**Published:** 2023-02-13

**Authors:** Jing Wang, Yu Chen, Huimin Chen, Long Hua, Jun Wang, Yuelong Jin, Lianping He, Yan Chen, Yingshui Yao

**Affiliations:** ^1^School of Public Health, Wannan Medical College, Wuhu, China; ^2^School of Medicine, Taizhou University, Jiaojiang, China; ^3^Department of Clinical Medicine, Anhui College of Traditional Chinese Medicine, Wuhu, China

**Keywords:** COVID-19, college students, coping strategies, social support, depression, parent-child relationship

## Abstract

**Objective:**

According to the WHO, compared to before the COVID-19 pandemic, young people showed a significant increase in depressive symptoms. In light of the recent coronavirus pneumonia pandemic, this study was conducted to determine how social support, coping style, parent-child relationships, and depression are associated. We investigated how these factors interacted and affected the prevalence of depression during this challenging and unheard-of time. Our research may help both individuals and healthcare professionals better comprehend and assist those who are coping with the pandemic’s psychological effects.

**Design and main outcome measures:**

3,763 students from a medical college in Anhui Province were investigated with Social Support Rate Scale, Trait Coping Style Questionnaire, and Self-rating Depression Scale.

**Results:**

When the pandemic situation was normalizing, social support was associated with depression and the coping style of college students (*p <* 0.01). During the period of pandemic normalization, the parent–child relationship moderated the relationship between social support and positive coping (*t* = −2.45, *p <* 0.05); the parent–child relationship moderated the relationship between social support and negative coping (*t* = −4.29, *p <* 0.01); and the parent–child relationship moderated the association between negative coping and depression (*t* = 2.08, *p <* 0.05).

**Conclusion:**

Social support has an impact on depression in the period of the regular prevention and control of COVID-19 through the mediating role of coping style and the moderating effect of the parent–child relationship.

## Introduction

The COVID-19 pandemic is associated with extremely high levels of psychological anguish, which, in many cases, would surpass the clinical relevance criteria (Xiong et al., 2020). This distress is frequently severe enough to require expert therapeutic attention. More study is needed to fully understand the pandemic’s psychological impact and to create suitable solutions to assist individuals impacted.

The likelihood of infection and mortality, loneliness, financial difficulties, and governmental regulations are the main elements causing this problem. According to surveys by Islam et al. (2020) and Qanash et al. (2020), college students are now more likely to experience a higher level of depression than they were before the COVID-19 pandemic. According to Deng’s study (Deng et al., 2021), different prevalence values apply, for example, there are geographic areas, diagnostic standards, educational attainment, undergraduate year of study, monetary status, living arrangements, and gender.

High levels of social support and affluence have been demonstrated in several studies to be protective factors against psychological illnesses (Lotzin et al., 2021). Utilization of resources, sources of support, and social support functions make up the bulk of the concept of social support. The presentation’s evidence (Cobb, 1976) demonstrates time and time again that social support is protective throughout a person’s life cycle and that it can shield those who are in danger from a range of dangers. Coping refers to the cognitive and behavioral effort of an individual to realize that their surroundings may impose an unbearable burden on themselves. Data show that social support can significantly influence coping styles, with groups with higher levels of social support responding to pressure from all sides more positively and maturely (Sun et al., 2020). After reviewing the data, it is found that many scholars regard coping style as a moderating effect after stress. In addition to coping style and social support having a direct impact on the victim’s adjustment, the importance of parental influence is also emphasized.

At the same time, according to the data (Zhu et al., 2020), there is a significant correlation between an individual’s social support and coping style after trauma. Social support was found to be significantly associated with an elevated risk for depression and poorer sleep quality (Grey et al., 2020). Research shows that individuals with positive coping are less likely to suffer from depression than those with negative coping (Budimir et al., 2021).

Structural equation modeling analysis showed that social support significantly predicted coping styles, both positively predicted student depression, respectively, and social support indirectly predicted college student depression through the mediating role of coping styles (Falgares et al., 2019). Due to differences in parent–child relationships, college students deal with problems differently in the face of stress, with students with discordant parent–child relationships suffering from psychological disorders such as anxiety, depression, and loneliness tendencies higher than college students with harmonious parent–child relationships. It has been suggested that the quality of the relationship between parents and college students may influence the effectiveness of coping styles in managing depression (Russell et al., 2020).

**Figure 1:**
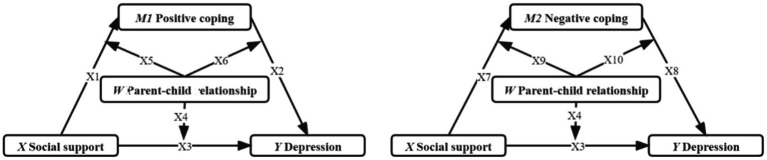
Hypothetical model: moderating mediator model.

A mediation model with moderating variables involved is built with social support as the independent variable (*X*), college student depression as the dependent variable(*Y*), coping style as the mediator mediating variable (*M1* & *M2*), and parent–child relationship as the Moderating variable (*W*) (Figure 1).

The purpose was to explore the relationship between depression and social support, the mediating role of coping style, and the moderating role of the parent–child relationship in this process. Meanwhile, the results of the study provide a valid reference for psychological interventions to improve intervention and coping among college students during the normalization of COVID-19 prevention and control.

## Methods

### Sample

Cross-sectional research was used in this study. The inclusion criteria were college students: (1) who were currently enrolled in universities, (2) who were staying in the epidemic areas of Mainland China during the outbreak of COVID-19, and (3) returning to school for the first time after a lockdown and home isolation. The exclusion criteria were students who showed regularity in responses (i.e., cases whose responses were all constant in the scales). After excluding cases that did not meet the inclusion criteria (*n* = 121), our final sample consisted of 3,763 individuals.

### Measurement

#### Demographics

The demographic characteristics included gender, age, school years (Junior or Senior), parent–child relationship (e.g., Poor, Average and Excellent), and current residence (Countryside or Non-rural).

#### Trait coping style questionnaire

The Trait Coping Style Questionnaire was developed by Jiang Qianjin (Zhiqun and Li, 1999) in 1999. It has 20 items that describe coping qualities, comprising two dimensions of positive coping (PC) and negative coping (NC), each with 10 items. The answers to each item’s questions range from “absolutely yes” to “certainly no.” The scale has a total score of 100 and is based on a 5-point Likert scale. The scale was designed to capture the individuals’ attitudes and behaviors, both positive and negative, in the face of challenges and failures. Each item is completed by subjects based on how well they do in the majority of scenarios. The scale was completed by 3,763 participants in total, and the NC and PC Cronbach’s coefficients were 0.83 and 0.66, respectively, demonstrating the excellent reliability of the survey.

#### Self-rating depression scale

The Depression Self-Rating Scale (SDS), created by Zung (Dunstan et al., 2017), has 20 items that range in difficulty from 1 to 4, with a standard score equal to the integer portion of the crude score (1.25). It measures depression status from the preceding week and seeks to identify patients who may be depressed. The standard score is the primary statistical indicator of the SDS, and the total scores across all items are regarded as crude scores that must be rounded by subsequent conversions because they cannot be utilized directly. The average result is attained. The elements were categorized as mild depression (53–62 points), moderate depression (63–72 points), and significant depression (73 points) using a four-level scale (1–4 points/items), with a score of 53 being positive for depression. The scale was completed by 3,763 participants in total. Cronbach’s alpha for the scale was 0.874, and the content validity was 0.853, with good internal consistency.

#### Social support rate scale

The Social Support Rating Scale (SSRS), created by Xiao (Shuiyuan, 1994; Boey, 1999), is a 10-point multi-axis scale with three parts: objective support (sum of scores for items 2, 6, and 7), subjective support (sum of scores for items 1, 3, 4, and 5), and utilization of support (sum of scores for items 8, 9, and 10). Each item receives a value between 0 and 4, with higher ratings denoting greater social support. On the scale, a total score of under 22 denotes a low level of social support, 23 to 44, a medium level, and 45 to 66, a high level. A total of 3,763 participants completed the scale in this study. With good reliability and validity, the scale has been consistently verified in the Chinese population, with Cronbach’s alphas for the overall scale, as well as 0.75, 0.79, and 0.77 for the three dimensions, respectively.

### Procedure

By using a stratified cluster sampling strategy from September to October 2020. Before beginning the questionnaire, which was voluntary, the first page of the document provided pertinent details about the study and its objectives. By checking a box, participants indicated their understanding of the survey’s terms and conditions. Before the subjects filled out the questionnaire, the researchers used established procedures to explain the study’s aim, content, and significance to participants. They also signed an agreement to keep the participants’ personal information private and collect their informed consent. They were told that their responses would be anonymized and asked to respond in good faith. The Wanan Medical College Ethics Committee gave its approval to this study (Ref. LL-2020BH01).

### Statistical analysis

The data were entered using EpiData 3.1, and statistical analysis was conducted using SPSS 26.0. Measures were expressed as (Mean ± SD), tests and control deviations were using Harman’s one-way test, descriptive statistical analysis for general demographic characteristics, correlation analysis to test for mediating effects, and regression analysis using the SPSS macro program Process plug-in created by Hayes for the mediated models with moderation (Hayes and Rockwood, 2017). The test’s alpha value was 0.05.

## Results

### Common method variance

All questionnaire items were put through exploratory factor analysis using Harman’s one-way test, and the analysis indicated only 13.38% of the variation could be explained by the first (biggest) common factor, which is considerably less than the usually accepted critical criteria of 40%. As a result, this study had no significant common variance bias.

### Analysis of general demographic characteristics

In total, 3,763 returning college students were chosen for the study as part of the normalization of the prevention and control of COVID-19 at a university in Anhui. Among these, 1920 females and 1843 males, with an average age of (18.92 ± 1.32) years; 64.50% of lower-year students and 35.50% of upper-year students, were recruited (Table 1).

**Table 1:** Descriptive of general demographic characteristics of returned college students (*n* = 3,763).

Variable	Groups	Frequency	Percent	Mean	SD
Gender	Male	1843	49.00	1.51	0.50
	Female	1920	51.00		
Grade	Junior	2,428	64.50	1.35	0.48
	Senior	1,335	35.50		
Age	17	393	10.40	18.92	1. 32
	18	1,344	35.70		
	19	920	24.40		
	20	588	15.60		
	21	324	8.60		
	22	194	5.20		
Parent–child relationship	Poor	151	4.00	2.78	0.50
	Average	513	13.60		
	Excellent	3,099	82.40		
Location	Countryside	1821	48.40	1.52	0.50
	Non-rural	1942	51.60		

### The relationship between social support and coping styles and depression levels was analyzed using bivariate analyses

Table 2 shows the Means, SDs, and Pearson correlations for all variables. The descriptive findings revealed that positive coping (Mean = 32.83, SD = 6.18), negative coping (Mean = 26.65, SD = 6.64), depression (Mean = 47.17, SD = 9.97), and social support (Mean = 37.92, SD = 5.60). The regression analysis revealed that social support was inversely related to depression (*r* = −0.328, *p* < 0.01), positively connected with positive coping (*r* = 0.296, *p* < 0.01), and negatively connected with negative coping (*r* = −0.290, *p* < 0.01). Additionally, there was a negative correlation between positive coping and depression (*r* = −0.451, *p* < 0.01) and a positive correlation between negative coping and depression (*r* = 0.390, *p* < 0.01).

**Table 2:** Pearson’s correlation coefficient between social support, coping styles, and depression (*n* = 3,763).

	Mean	SE	Social support	Depression	Positive coping	Negative coping
Social support	37.92	5.60	1.000	–	–	–
Depression	47.17	9.97	−0.328^**^	1.000	–	–
Positive coping	32.83	6.18	0.296^**^	−0.451^**^	1.000	
Negative coping	26.65	6.64	−0.290^**^	0.390^**^	−0.168^**^	1.000

^**^p < 0.01.

### Coping style as a mediating variable between the variables

The mediation model was tested in two steps in this study, by the methodology suggested by Zhonglin and Baojuan (2014). First, 5,000 bootstrap samples were used to test for mediating effects and Model 4 in PROCESS 3.4. Using social support as the *X* variable, depression as the *Y* variable, positive coping style and negative coping style as the *M1* and *M2* variables, and age and gender as the control variables, were chosen using the SPSS macro program Process created by Hayes and Rockwood (2017).

The analysis indicates that social support significantly impacted depression, positive coping, and both negative and positive coping (*p* < 0.01), and that the effects continued unabated even after adding the mediating variable of coping style. Additionally, none of the bootstrap’s 95% confidence intervals contained 0, further demonstrating how well the mediation model fit. The relative efficiency of the direct effect was 41.41%, while the relative efficiency of the mediating effect was positive = 31.95% and negative = 26.62%, respectively (Tables 3, 4).

**Table 3:** Mediated variable testing: coping style.

Variable	Depression		Depression		Positive coping		Negative coping	
	*t*	*p*	*t*	*p*	*t*	*p*	*t*	*p*
Gender	−1.379	0.168	−0.571	0.568	4.137	<0.01	7.323	<0.01
Age	0.169	0.866	−0.244	0.808	−2.697	0.007	−4.677	<0.01
Social support	−9.292	<0.01	−21.191	<0.01	18.650	<0.01	−19.249	<0.01
Negative coping	20.491	<0.01	–	–	–	–	–	
Positive coping	−25.389	<0.01						
*R^2^*	0.321		0.108		0.094		0.103	
*F*	355.917		150.886		129.270		144.037	

All variables in the model are brought into the regression equation with standardized variables.

**Table 4:** Tests of the total effect, direct effect, and mediated effect variables.

	Effect	BootSE	BootLLCI	BootULCI	Effect proportion
Positive coping	−0.186	0.014	−0.216	−0.159	0.320
Negative coping	−0.155	0.012	−0.181	−0.132	0.266
Direct effect	−0.242	0.026	−0.293	−0.191	0.414
Total effect	−0.583	0.028	−0.637	−0.529	

BootSE, BootllCI, and BootulCI refer to the upper and lower limits of 95% confidence interval and standard error of indirect effects estimated by deviation corrected percentile bootstrap method.

### The moderation effect of the parent–child relationship on depression

**Figure 2:**
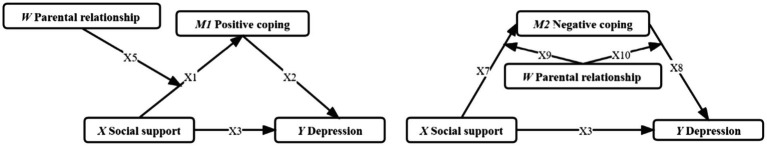
Significant pathways of the parent–child relationship in the moderated model.

To test for significant mediating models, the second step is to add moderating variables. The parent–child relationship was added as a moderating variable in this study using model 59, while *X*, *Y*, and control variables were kept constant from the first step (Figure 2) The results show that, first, the parent–child relationship moderated the relationship between social support and positive coping (*t* = −2.45, *p* < 0.05); second, the parent–child relationship moderated the relationship between social support and negative coping (*t* = −4.29, *p* < 0.01); and parent–child relationship moderated the association between negative coping and depression (*t* = 2.08, *p* < 0.05). Additionally, the findings reveal that the upper and lower limits of the 95% confidence intervals for the Bootstrap method do not contain zero, supporting the idea that the parent–child relationship, a moderating variable, is important in this model (Tables 5, 6).

**Table 5:** Moderated mediation model test.

	Positive coping			Negative coping			Depression		
	*β*	SE	*t*	*β*	SE	*t*	*β*	SE	*t*
Positive coping							−0.56	0.02	−24.39^**^
Negative coping							0.44	0.02	20.44^**^
Positive coping × Parent–child relationship							0.04	0.02	1.72
Negative coping × Parent–child relationship							0.05	0.02	2.08^*^
Social support × Parent–child relationship	−0.04	0.02	−2.45^*^	−0.08	0.02	−4.29^**^	0.03	0.03	1.02
Social support	0.29	0.02	16.14^**^	−0.34	0.02	−17.64^**^	−0.18	0.03	−6.90^**^
Parent–child relationship	0.61	0.11	5.35^**^	−0.48	0.12	−3.94^**^	−1.28	0.16	−7.95^**^
Gender	0.76	0.19	3.98^**^	1.46	0.20	7.14^**^	−0.35	0.27	−1.29
Age	−0.21	0.07	−2.81^**^	−0.37	0.08	−4.74^**^	0.04	0.10	0.42

^*^p < 0.05; ^**^p < 0.01; α = 0.05.

**Table 6:** The coping style adapts to the moderating influence of parent–child relationships.

Parent–child relationship	Effect	BootSE	BootLLCI	BootULCI
*Positive coping*				
*M* − 1SD	−0.195	0.020	−0.235	−0.158
*M*	−0.161	0.014	−0.189	−0.133
*M* + 1SD	−0.134	0.017	−0.169	−0.101
*Negative coping*				
*M* − 1SD	−0.104	0.015	−0.135	−0.076
*M*	−0.147	0.012	−0.172	−0.125
*M* + 1SD	−0.189	0.018	−0.225	−0.153

BootSE, BootllCI, and BootulCI refer to the upper and lower limits of 95% confidence interval and standard error of indirect effects estimated by deviation corrected percentile bootstrap method.

### Simple slope plots

**Figure 3:**
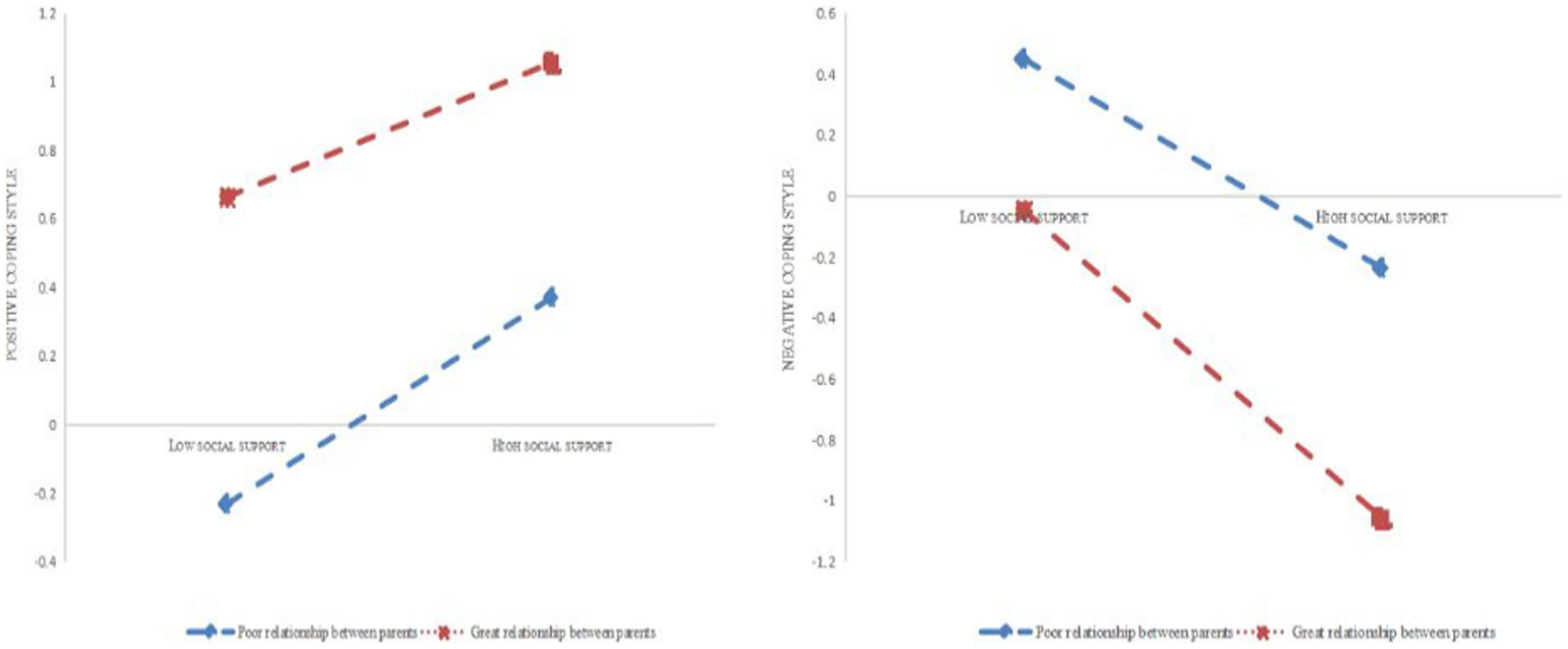
The moderating role of the parent–child relationship in social support and coping style.

**Figure 4:**
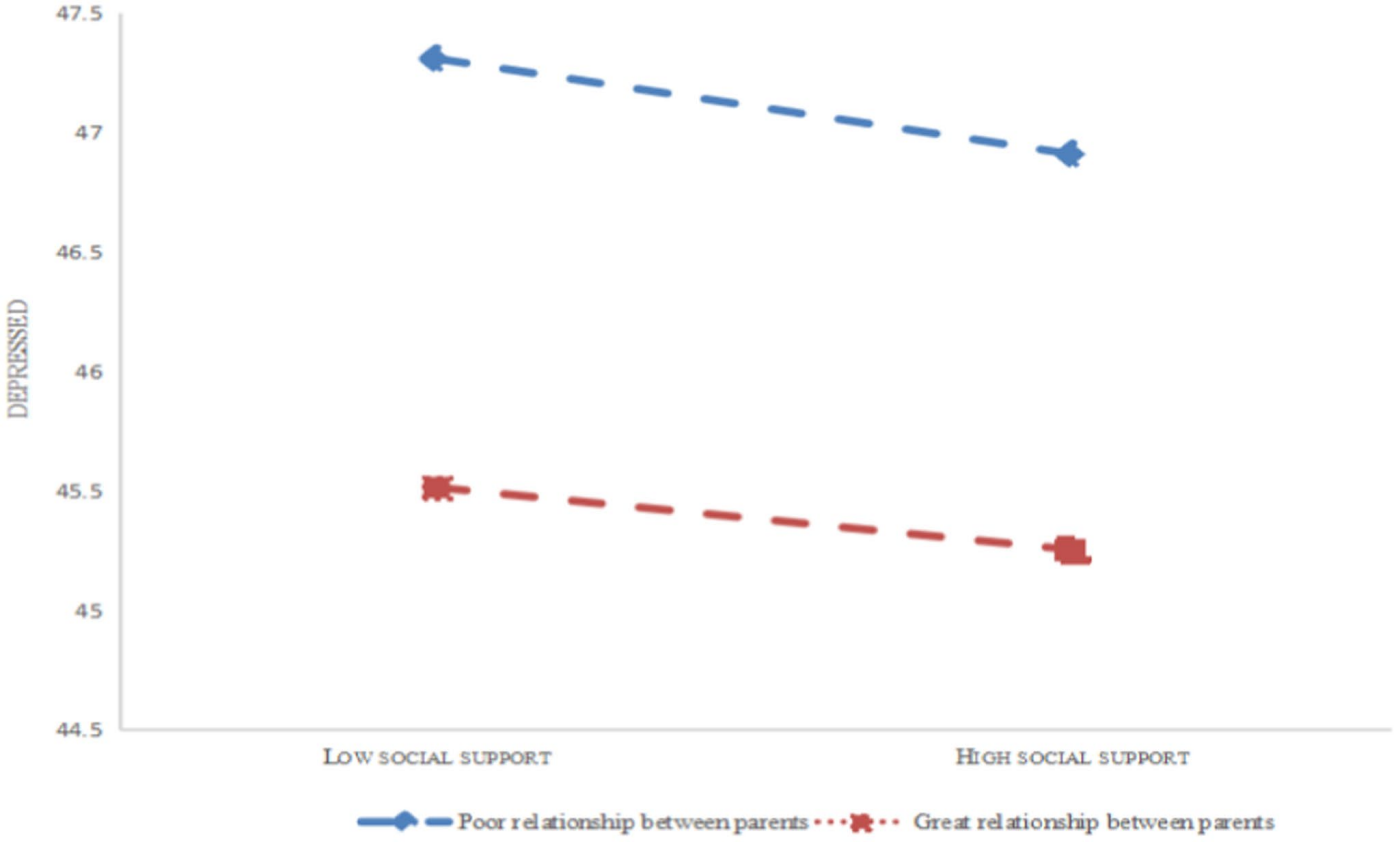
The moderating role of the parent–child relationship in social support and depression of college students.

With positive coping style functioning as the mediating variable, further simple slope plot analysis indicates (Figures 3, 4) that the level of social support is significantly higher for people who had good parenting than for people who had poor parenting. That is, at the same level of social support and coping styles, the slope was higher for good parent–child relationships than for poor parent–child relationships. In contrast, with negative coping style acting as the mediating variable, the hypothesis of a moderating effect of parents is still completely accurate. Similar to the above, at the same level of social support, those with good parent–child relationships had significantly lower rates of depression than those with poor parent–child relationships.

## Discussion

The results of this study are interpreted in three main aspects. In terms of research correlation, social support, coping style, and depression are all linked. In additional analyses, even after controlling for the relevant third variables such as college students’ gender and age. It was discovered that both positive and negative coping functioned as mediating various that mediated the relationship between social support and depression.

### The mediating role of coping styles

The current study discovered that social support had a significant negative predictive effect on depression among college students. According to EbruIkiz and SaviCakar (2010), College students’ mental health can suffer from a lack of social support, which can also lead to the onset of depression among college students (MacMillan et al., 2021). During the teenage years (Hefner and Eisenberg, 2009), there is a great opportunity for personal expansion and psychological maturity, and having supportive relationships with others can help college students manage the anxieties brought on by the coronavirus pandemic while having solid backing from the external environment bolsters their confidence in tackling issues. When adolescents are provided with a supportive environment, the risk of emotional and behavioral issues and depression can be significantly decreased (Lin et al., 2020). Novel coronavirus infections and their associated control measures, college students entering an online learning environment face multiple mental health issues due to the sharp deviation from their normal lifestyle, which may be further exacerbated by policy-based blockages that prevent students from receiving on-site mental health counseling and emotional support provided from family and friends (Deng et al., 2021). The resulting psychological distress may further develop into co-occurring disorders, such as decreased self-esteem or self-efficacy (Saleh et al., 2017); if left unattended or untreated, these co-occurring disorders may lead to serious consequences, such as suicidal thoughts or other mental illnesses (Arria et al., 2009). According to G’s research (Gariépy et al., 2016), parental social support and the ability of parents to provide for their children’s basic needs, including emotional and material ones, are both strongly related to depression in adolescents. Support from parents and family members is a protective factor against depression in adolescents, and this support provides more benefits than any other source, peer social support also proved to be crucial for college students’ development when parental social support was absent. Furthermore, the effects of post-traumatic stress during an epidemic should not be underestimated (Sher, 2020), particularly if the person is surrounded by people who have died or contracted the virus, making the perceived threat to their life even greater. Currently, medical insurance from the state is an important strategy to ease college students’ psychological crisis, especially when the person is surrounded by people who have either died from the virus or have already contracted it, making the perceived threat to life even greater. The state’s provision of medical insurance is currently a crucial step in addressing college students’ psychological distress, especially because as more Chinese people have received their COVID-19 vaccinations, there is a markedly decreased risk that the group will develop infections or require hospitalization related to the disease (Mohamed et al., 2022).

The positive coping style had a significant negative predictive effect on college students’ depression. The study (Lee and Cho, 2021) revealed that cognitive behavioral therapy for depression aims to ease the pain of the cognitive revision process, readjust the patient’s perception of reality, help someone reconstruct correct cognition, objective evaluation, affirm himself or herself, and enhance self-confidence. Benefit finding was recognized as one of the key protective factors against depression, anxiety, and stress in another study (Tang et al., 2021). The child’s belief that home isolation would increase time spent with parents and personal activities explains this. Optimistic students are more likely to experience negative emotions than pessimistic students (Xie et al., 2020). This necessitates the use of cognitive-behavioral therapy by groups (school, family, and clinical care) to raise students’ awareness of the pandemic and alleviate their depressed mood (Vagni et al., 2020).

There has been little research in China on coping strategies, particularly crisis coping. However, U.S. data report effective coping strategies for groups in the face of increased deaths due to COVID-19 (DeDonno et al., 2022), predominantly acceptance, self-distraction, and use of emotional support (including emotional support from family and peers as studied previously). Stewart’s coping theory states that social support should be identified as one of the coping resources (Kassam, 2019). These findings support the main effects model of coping style. Brittney Riedel’s result showed (Riedel et al., 2021) that the implementation of healthy coping skills and therapeutic interventions will allow individuals to escape the damage caused by the new coronary outbreak. In other words, college students are more likely to take the initiative to adopt protective coping strategies, actively cooperate with medical and nursing staff in all treatment, and maintain their physical, psychological, and social health at a higher level the more social support they receive in the face of stressful events sparked by the new coronary pneumonia pandemic. On the other hand, the psychological stress brought on by the stressful event will be increased if they passively adopt a yielding coping strategy (Min et al., 2019; Ekram et al., 2022). These two points explain exactly how college students may spontaneously accept help from the external environment and implement self-transference of pain in the face of the new crown epidemic, while the other point explains how college students may choose to passively accept the new crown epidemic in the face of the numerous harms it brings.

### The moderating effect of the parent–child relationship

The parent–child relationship showed up to influence the association between social support and positive coping (PathX5), as shown by the results of the model analysis that took into account that relationship as a moderating factor. Both the relationship between negative coping and depression (PathX8) and the relationship between social support and negative coping (PathX7) were moderated by the parent–child relationship (Figure 4).

Previous research (Grunin et al., 2021) has mainly concentrated on how family factors, such as rearing styles and parental child abuse, affect the development of adolescents’ mental and physical health (including bullying, aggressive behavior, depression, anxiety, etc.). According to Chorot’s research (Chorot et al., 2017), insecure attachment is linked to depressive symptoms in children and higher levels of depression in adolescent development, and parenting style has a significant impact on adolescent depression. According to Bögel’s findings (Bögels and Brechman-Toussaint, 2006), among many other family-related factors, the relationship with parents has a significant impact on adolescent mental health. Lopez et al. (2012) showed a correlation between early parental abuse and coping skills in adolescents. This is consistent with the results of our study. Low levels of parent–child relationships are more likely to result in unfavorable ways of handling stress from the environment, which raises the risk of depression. But the parent–child relationship as a moderating effect was not studied during the pandemic.

The findings (Xu et al., 2022) indicate that a positive relationship between parents and children raises the level of social support for the person, which improves their psychological health and reduces their risk of mental health issues. College students in this study experienced numerous localized outbreaks of the pandemic, social isolation, and fear of disease transmission to the point where they were more hopeless, anxious, nervous, depressed, and irritable than ever before. Furthermore, it’s critical to consider the parent–child relationship’s moral and material support in this situation.

The positive parent–child relationship can help students cope with depression. According to a study (Tang et al., 2021), Parent–child discussions are another protective factor in reducing depressive symptoms in adolescents during the pandemic. The quality of the parent–child relationship in the family system must be improved by parents giving their children unconditionally positive attention, increased emotional support, and spending more time with them overall to improve the parent–child relationship within the family unit. Raising the child’s self-esteem, cultivating emotional fortitude, and enhancing their capacity to enhance child’s capacity to transform negative feelings into positive ones when facing challenging circumstances. This will lessen the likelihood of childhood depression (Janssens et al., 2021). Research shows (Zhang and Wills, 2016) that Chinese parents tend to use supportive behaviors rather than verbal expressions to convey care to their children. Therefore, there is a particular need to encourage Chinese parents to discuss life events more frequently with their children to promote their children’s mental health during public health crises.

Our investigation revealed that supportive parental resources influence college students’ depressed moods through a variety of pathways, including both the mediating role of coping styles and the moderating role of parent–child relationships. The presence of strong parental backing assures college students to effectively manage the effects of the pandemic, particularly when social distancing measures are in place. Emotional backing and financial stability from their families are especially crucial during this time. This highlights the significance of familial influence in aiding college students to manage through trying times, particularly parental backing.

## Conclusion

In conclusion, this study evaluated the prevalence of depression among college students during COVID-19 and discovered through model testing that coping style as well as parental relationships had an impact on the occurrence of depression.

## Limitations and future research

The main limitations of our study are (1) although the cross-sectional design of our study prevented us from demonstrating how these variables changed over time, our follow-up cohort study is already underway; (2) Data collection is yet another restriction. Each participant in this study completed their questionnaire, which could have led to subjectivity or reliability bias. The participants who responded may have been limited by the technical tools used for whole cohort sampling, which resulted in a population that was not completely represented. To include different types of college students, we will therefore use a more thorough collection strategy in subsequent studies (e.g., major, grade, region, etc.). In addition to this, another concept that needs to be studied is the emo-sensory load of the environment and people. COVID-19 can negativize individuals’ emotions making them behave differently, which needs to be examined in further studies.

## Data Sharing

Data generated or analyzed during the study are available from the corresponding author by request.

## Data availability statement

The original contributions presented in the study are included in the article/supplementary material, further inquiries can be directed to the corresponding authors.

## Ethics statement

The studies involving human participants were reviewed and approved by The School of public health of Wannan Medical College. The patients/participants provided their written informed consent to participate in this study.

## Author contributions

YaC and YY: conceived and designed the study. JiW, YuC, HC, LoH, and JuW: collected the data. JiW and YY: interpreted the data. JiW and YuC: wrote the first draft of the manuscript. LiH, YaC, and YJ: modified the manuscript. LiH and YaC: reviewed the manuscript. All authors contributed to the article and approved the submitted version.

## Funding

This study was supported by the Fifth Batch of Talents Selected under the Special Support Plan in Anhui Provence (Organization Department of Anhui provincial Party committee, [2019] No.14, T000516); Major natural science research Projects in Universities of Anhui Province (no. KJ2020ZD69).

## Conflict of interest

The authors declare that the research was conducted in the absence of any commercial or financial relationships that could be construed as a potential conflict of interest.

## Publisher’s note

All claims expressed in this article are solely those of the authors and do not necessarily represent those of their affiliated organizations, or those of the publisher, the editors and the reviewers. Any product that may be evaluated in this article, or claim that may be made by its manufacturer, is not guaranteed or endorsed by the publisher.

## References

[ref3] Arria A. M. O'Grady K. E. Caldeira K. M. Vincent K. B. Wilcox H. C. Wish E. D. (2009). Suicide ideation among college students: a multivariate analysis. Arch. Suicide Res. 13, 230–246. doi: 10.1080/13811110903044351, PMID: 19590997 PMC2709750

[ref4] Boey K. W. (1999). Help-seeking preference of college students in urban China after the implementation of the "open-door" policy. Int. J. Soc. Psychiatry 45, 104–116. doi: 10.1177/002076409904500203, PMID: 10443253

[ref5] Bögels S. M. Brechman-Toussaint M. L. (2006). Family issues in child anxiety: attachment, family functioning, parental rearing and beliefs. Clin. Psychol. Rev. 26, 834–856. doi: 10.1016/j.cpr.2005.08.001, PMID: 16473441

[ref6] Budimir S. Probst T. Pieh C. (2021). Coping strategies and mental health during COVID-19 lockdown. J. Ment. Health 30, 156–163. doi: 10.1080/09638237.2021.1875412 33502917

[ref7] Chorot P. Valiente R. M. Magaz A. M. Santed M. A. Sandin B. (2017). Perceived parental child rearing and attachment as predictors of anxiety and depressive disorder symptoms in children: the mediational role of attachment. Psychiatry Res. 253, 287–295. doi: 10.1016/j.psychres.2017.04.015, PMID: 28411577

[ref8] Cobb S. (1976). Presidential Address-1976. Social support as a moderator of life stress. Psychosom. Med. 38, 300–314. doi: 10.1097/00006842-197609000-00003 981490

[ref9] DeDonno M. A. Ferris A. H. Molnar A. Haire H. M. Sule S. S. Hennekens C. H.. (2022). Perceptions, coping strategies, and mental health of residents during COVID-19. South Med. J. 115, 717–721. doi: 10.14423/smj.0000000000001439 36055661 PMC9426312

[ref10] Deng J. Zhou F. Hou W. Silver Z. Wong C. Y. Chang O.. (2021). The prevalence of depressive symptoms, anxiety symptoms and sleep disturbance in higher education students during the COVID-19 pandemic: a systematic review and meta-analysis. Psychiatry Res. 301:113863. doi: 10.1016/j.psychres.2021.113863, PMID: 33984824 PMC9225824

[ref11] Dunstan D. A. Scott N. Todd A. K. (2017). Screening for anxiety and depression: reassessing the utility of the Zung scales. BMC Psychiatry 17:329. doi: 10.1186/s12888-017-1489-6, PMID: 28886698 PMC5591521

[ref12] EbruIkiz F. SaviCakar F. (2010). Perceived social support and self-esteem in adolescence. Procedia Soc. Behav. Sci. 5, 2338–2342. doi: 10.1016/j.sbspro.2010.07.460

[ref13] Ekram M. Cao Y. Zhang C. (2022). The role of hope level and disease coping style in mediating the chain between social support and quality of life in breast cancer patients. Med. Soc. 35:95-100+105. doi: 10.13723/j.yxysh.2022.11.018

[ref14] Falgares G. Lo Gioco A. Verrocchio M. C. Marchetti D. (2019). Anxiety and depression among adult amputees: the role of attachment insecurity, coping strategies and social support. Psychol. Health Med. 24, 281–293. doi: 10.1080/13548506.2018.1529324, PMID: 30299156

[ref15] Gariépy G. Honkaniemi H. Quesnel-Vallée A. (2016). Social support and protection from depression: systematic review of current findings in Western countries. Br. J. Psychiatry 209, 284–293. doi: 10.1192/bjp.bp.115.169094 27445355

[ref16] Grey I. Arora T. Thomas J. Saneh A. Tohme P. Abi-Habib R. (2020). The role of perceived social support on depression and sleep during the COVID-19 pandemic. Psychiatry Res. 293:113452. doi: 10.1016/j.psychres.2020.113452, PMID: 32977047 PMC7500407

[ref17] Grunin L. Yu G. Cohen S. S. (2021). The relationship between youth cyberbullying behaviors and their perceptions of parental emotional support. Int. J. Bullying Prev. 3, 227–239. doi: 10.1007/s42380-020-00080-5, PMID: 33005875 PMC7465883

[ref18] Hayes A. F. Rockwood N. J. (2017). Regression-based statistical mediation and moderation analysis in clinical research: observations, recommendations, and implementation. Behav. Res. Ther. 98, 39–57. doi: 10.1016/j.brat.2016.11.001, PMID: 27865431

[ref19] Hefner J. Eisenberg D. (2009). Social support and mental health among college students. Am. J. Orthopsychiatry 79, 491–499. doi: 10.1037/a0016918 20099940

[ref20] Islam M. S. Sujan M. S. H. Tasnim R. Sikder M. T. Potenza M. N. van Os J. (2020). Psychological responses during the COVID-19 outbreak among university students in Bangladesh. PLoS One 15:e0245083. doi: 10.1371/journal.pone.0245083, PMID: 33382862 PMC7775049

[ref21] Janssens J. J. Achterhof R. Lafit G. Bamps E. Hagemann N. Hiekkaranta A. P.. (2021). The impact of COVID-19 on Adolescents' daily lives: the role of parent-child relationship quality. J. Res. Adolesc. 31, 623–644. doi: 10.1111/jora.12657, PMID: 34448305 PMC8646476

[ref22] Kassam S. (2019). Understanding experiences of social support as coping resources among immigrant and refugee women with postpartum depression: an integrative literature review. Issues Ment. Health Nurs. 40, 999–1011. doi: 10.1080/01612840.2019.1585493, PMID: 31070499

[ref23] Lee S. H. Cho S. J. (2021). Cognitive behavioral therapy and mindfulness-based cognitive therapy for depressive disorders. Adv. Exp. Med. Biol. 1305, 295–310. doi: 10.1007/978-981-33-6044-0_16 33834406

[ref24] Lin J. Su Y. Lv X. Liu Q. Wang G. Wei J.. (2020). Perceived stressfulness mediates the effects of subjective social support and negative coping style on suicide risk in Chinese patients with major depressive disorder. J. Affect. Disord. 265, 32–38. doi: 10.1016/j.jad.2020.01.026, PMID: 31959583

[ref25] Lopez C. M. Begle A. M. Dumas J. E. de Arellano M. A. (2012). Parental child abuse potential and subsequent coping competence in disadvantaged preschool children: moderating effects of sex and ethnicity. Child Abuse Negl. 36, 226–235. doi: 10.1016/j.chiabu.2011.10.012, PMID: 22425165 PMC7890930

[ref26] Lotzin A. Krause L. Acquarini E. Ajdukovic D. Ardino V. Arnberg F.. (2021). Risk and protective factors, stressors, and symptoms of adjustment disorder during the COVID-19 pandemic – first results of the ESTSS COVID-19 pan-European ADJUST study. Eur. J. Psychotraumatol. 12:1964197. doi: 10.1080/20008198.2021.1964197, PMID: 34992755 PMC8725769

[ref27] MacMillan K. K. Lewis A. J. Watson S. J. Bourke D. Galbally M. (2021). Maternal social support, depression and emotional availability in early mother-infant interaction: findings from a pregnancy cohort. J. Affect. Disord. 292, 757–765. doi: 10.1016/j.jad.2021.05.048, PMID: 34167025

[ref28] Min Y. Xiehong Z. Xia C. Shujuan S. Yiping H. Sai L. (2019). Effects of social support and coping styles on stigma of cervical cancer patients. Chin. J. Clin. Psych. 27, 1139–1143. doi: 10.16128/j.cnki.1005-3611.2019.06.013

[ref29] Mohamed K. Rzymski P. Islam M. S. Makuku R. Mushtaq A. Khan A.. (2022). COVID-19 vaccinations: the unknowns, challenges, and hopes. J. Med. Virol. 94, 1336–1349. doi: 10.1002/jmv.27487, PMID: 34845731 PMC9015467

[ref34] Qanash S. Al-Husayni F. Alemam S. Alqublan L. Alwafi E. Mufti H. N.. (2020). Psychological effects on health science students after implementation of COVID-19 quarantine and distance learning in Saudi Arabia. Cureus 12:e11767. doi: 10.7759/cureus.11767, PMID: 33409015 PMC7779124

[ref35] Riedel B. Horen S. R. Reynolds A. Hamidian Jahromi A. (2021). Mental health disorders in nurses during the COVID-19 pandemic: implications and coping strategies. Rev. Front. Public Health 9:707358. doi: 10.3389/fpubh.2021.707358, PMID: 34765579 PMC8575697

[ref36] Russell B. S. Hutchison M. Tambling R. Tomkunas A. J. Horton A. L. (2020). Initial challenges of caregiving during COVID-19: caregiver burden, mental health, and the parent-child relationship. Child Psychiatry Hum. Dev. 51, 671–682. doi: 10.1007/s10578-020-01037-x, PMID: 32749568 PMC7398861

[ref37] Saleh D. Camart N. Romo L. (2017). Predictors of stress in college students. Front. Psychol. 8:19. doi: 10.3389/fpsyg.2017.00019, PMID: 28179889 PMC5263159

[ref38] Sher L. (2020). The impact of the COVID-19 pandemic on suicide rates. QJM 113, 707–712. doi: 10.1093/qjmed/hcaa202, PMID: 32539153 PMC7313777

[ref39] Shuiyuan X. (1994). Theoretical foundations and research applications of the social support rating scale. J. Clin. Psychiatry 2, 98–100.

[ref40] Sun J. Harris K. Vazire S. (2020). Is well-being associated with the quantity and quality of social interactions? J. Pers. Soc. Psychol. 119, 1478–1496. doi: 10.1037/pspp0000272 31647273

[ref41] Tang S. Xiang M. Cheung T. Xiang Y. T. (2021). Mental health and its correlates among children and adolescents during COVID-19 school closure: the importance of parent-child discussion. J. Affect. Disord. 279, 353–360. doi: 10.1016/j.jad.2020.10.016, PMID: 33099049 PMC7550131

[ref42] Vagni M. Maiorano T. Giostra V. Pajardi D. (2020). Coping with COVID-19: emergency stress, secondary trauma and self-efficacy in healthcare and emergency Workers in Italy. Front. Psychol. 11:566912. doi: 10.3389/fpsyg.2020.566912, PMID: 33013603 PMC7494735

[ref43] Xie X. Xue Q. Zhou Y. Zhu K. Liu Q. Zhang J.. (2020). Mental health status among children in home confinement during the coronavirus disease 2019 outbreak in Hubei Province. China. JAMA Pediatr. 174, 898–900. doi: 10.1001/jamapediatrics.2020.1619, PMID: 32329784 PMC7182958

[ref44] Xiong J. Lipsitz O. Nasri F. Lui L. M. W. Gill H. Phan L.. (2020). Impact of COVID-19 pandemic on mental health in the general population: a systematic review. J. Affect. Disord. 277, 55–64. doi: 10.1016/j.jad.2020.08.001, PMID: 32799105 PMC7413844

[ref45] Xu W. Yanling L. Jie L. Chuanxing L. Lingzhen W. Hanyu Q. (2022). The effect of parent-child relationship on secondary school students' mental health: the chain mediating role of social support and psychological quality. Psychol. Dev. Educ. 38, 263–271. doi: 10.16187/j.cnki.issn1001-4918.2022.02.13

[ref46] Zhang Q. Wills M. (2016). A U.S.-Chinese comparison of affectionate communication in parent-child relationships. Commun. Res. Rep. 33, 317–323. doi: 10.1080/08824096.2016.1224166

[ref002] Zhiqun L. Li L. (1999). A study of college students’ coping styles. J. Shanxi Med. Univ. 2, 14–15.

[ref48] Zhonglin W. Baojuan Y. (2014). Mediated effects analysis: methods and model development. Adv. Psychol. Sci. 22, 731–745.

[ref49] Zhu W. Wei Y. Meng X. Li J. (2020). The mediation effects of coping style on the relationship between social support and anxiety in Chinese medical staff during COVID-19. BMC Health Serv. Res. 20:1007. doi: 10.1186/s12913-020-05871-6, PMID: 33148229 PMC7609823

